# Supplementary material for: MetaRibo-Seq measures translation in microbiomes
Source: Nat Commun. 2020 Jun 29;11:3268. doi: 10.1038/s41467-020-17081-z (PMC7324362; doi:10.1038/s41467-020-17081-z)
Supplement: Supplementary file 10 — Supplementary Data 7 [file 41467_2020_17081_MOESM10_ESM.zip › File2/Confidence_High_Taxonomy/144402_out.krona.html]

Javascript must be enabled to view this page.

members
magnitude
magnitudeUnassigned
count
unassigned
taxon
rank

144402\_out

2

superkingdom
2
2

2
phylum
976

200643
class
2

order
2
171549

815
2
family

2
genus
816


SRS063040\_contig\_number\_33227
1
species
818

371601
species
1

SRS893380\_contig\_number\_2216
